# Supplementary material for: Dual arginine recognition of LRRK2 phosphorylated Rab GTPases
Source: Biophys J. 2021 Apr 20;120(9):1846–55. doi: 10.1016/j.bpj.2021.03.030 (PMC8204342; doi:10.1016/j.bpj.2021.03.030)
Supplement: Document S1. Fig. S1 [file mmc1.pdf]

Biophysical Journal, Volume *120*

Supplemental information

Dual arginine recognition of LRRK2 phosphorylated Rab GTPases

Dieter Waschbüsch, Elena Purlyte, and Amir R. Khan

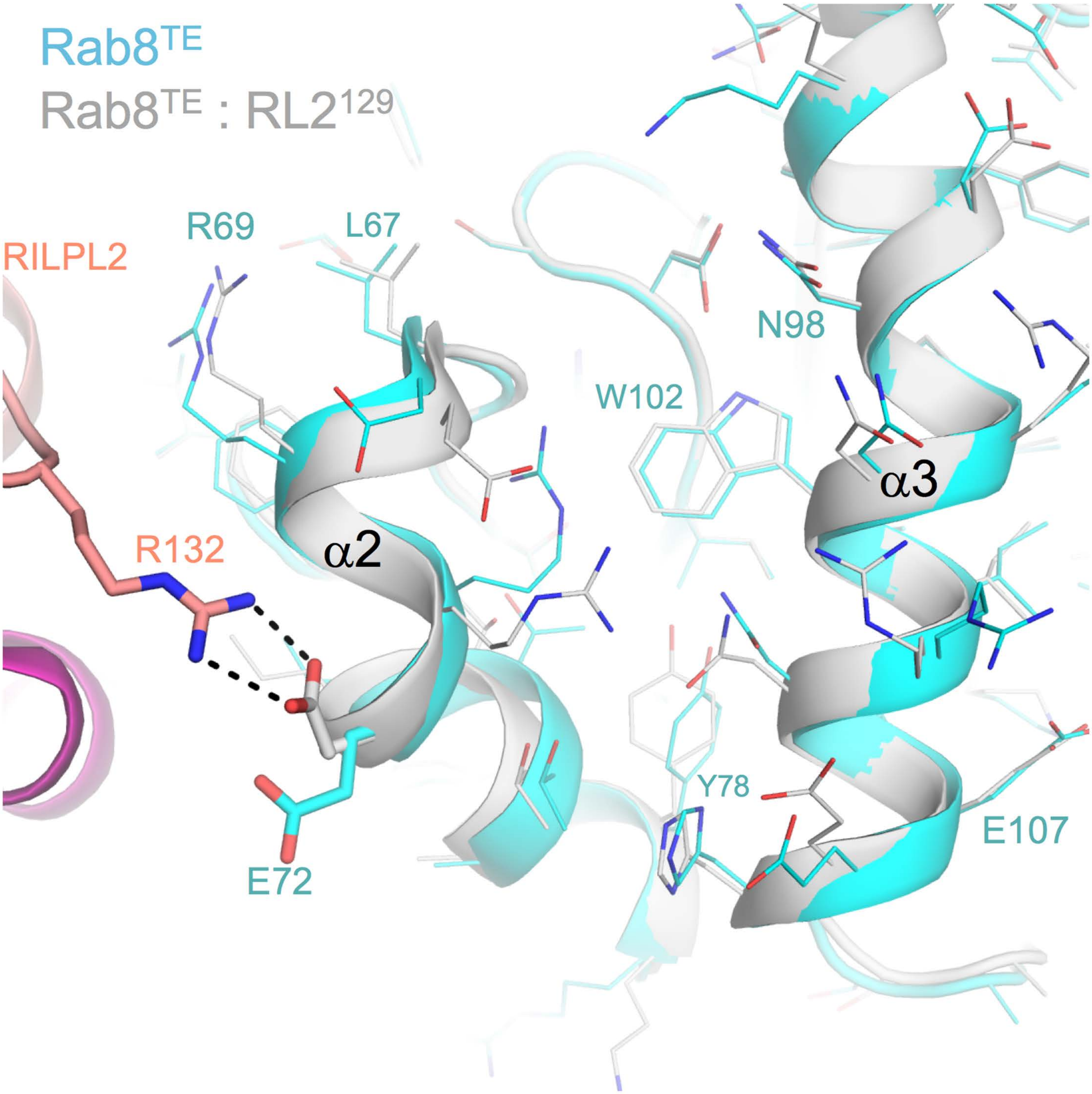
Rab8TE
Rab8TE : RL2129
R69
L67
RILPL2
N98
W102
α3
α2
R132
Y78
E107
E72

Figure S1
