# Supplementary material for: Dual arginine recognition of LRRK2 phosphorylated Rab GTPases
Source: Biophys J. 2021 Apr 20;120(9):1846–55. doi: 10.1016/j.bpj.2021.03.030 (PMC8204342; doi:10.1016/j.bpj.2021.03.030)
Supplement: Document S2. Article plus supporting material [file mmc3.pdf]

Biophysical Journal
Article

Dual arginine recognition of LRRK2 phosphorylated Rab GTPases

Dieter Waschbüsch,[1] Elena Purlyte,[2] and Amir R. Khan[3,*]
[1]School of Biochemistry and Immunology, Trinity College Dublin, Dublin, Ireland; [2]MRC Protein Phosphorylation and Ubiquitylation Unit, School of Life Sciences, University of Dundee, Dundee, United Kingdom; and [3]Division of Newborn Medicine, Boston Children's Hospital, Boston, Massachusetts

ABSTRACT Parkinson's-disease-associated LRRK2 is a multidomain Ser/Thr kinase that phosphorylates a subset of Rab GTPases to control their effector functions. Rab GTPases are the prime regulators of membrane trafficking in eukaryotic cells. Rabs exert their biological effects by recruitment of effector proteins to subcellular compartments via their Rab-binding domain (RBD). Effectors are modular and typically contain additional domains that regulate various aspects of vesicle formation, trafficking, fusion, and organelle dynamics. The RBD of effectors is typically an α-helical coiled coil that recognizes the GTP conformation of the switch 1 and switch 2 motifs of Rabs. LRRK2 phosphorylates Rab8a at T72 (pT72) of its switch 2 α-helix. This post-translational modification enables recruitment of RILPL2, an effector that regulates ciliogenesis in model cell lines. A newly identified RBD motif of RILPL2, termed the X-cap, has been shown to recognize the phosphate via direct interactions between an arginine residue (R132) and pT72 of Rab8a. Here, we show that a second distal arginine (R130) is also essential for phospho-Rab binding by RILPL2. Through structural, biophysical, and cellular studies, we find that R130 stabilizes the primary R132:pT72 salt bridge through favorable enthalpic contributions to the binding affinity. These findings may have implications for the mechanism by which LRRK2 activation leads to assembly of phospho-Rab complexes and subsequent control of their membrane trafficking functions in cells.

SIGNIFICANCE Parkinson's-disease-associated LRRK2 kinase phosphorylates Rab8a and Rab10 to subvert their membrane trafficking functions in the formation of primary cilia. Rab8a is phosphorylated at a threonine (pT72) and subsequently binds to a phosphospecific effector (RILPL2) via its C-terminal RH2 domain. Here, we show that recognition of pT72 involves a dual arginine motif involving R130 and R132 from the RH2 domain of RILPL2. Thermodynamic and structural studies suggest that the phosphospecific interactions are highly sensitive to the upstream region, which includes the N-terminal RH1 domain that binds to myosin Va. The dual arginine motif may facilitate the dynamic nature of membrane trafficking processes involving phosphorylation of Rabs.

INTRODUCTION

Rab GTPases belong to the Ras superfamily of GTP/GDP-binding molecular switches that regulate membrane trafficking in eukaryotic cells (1). GTP/GDP exchange factors (GEFs) convert Rabs into their GTP form and are involved in directing them onto distinct subcellular compartments via their prenylated C-terminal cysteine residues. The switch 1 and 2 regions of Rabs undergo local conformational changes during the nucleotide exchange that enable recruitment of GTP-specific cytosolic effector proteins (2). Rabs are turned off through the action of GTPase activating proteins (GAPs) that aid the hydrolysis of the γ-phosphate of GTP. In recent years, several Rab GTPases have been shown to be targets of protein kinases (3). Phosphorylation of Rabs has emerged as a mechanism for tuning Rab functions through control of the strength of their interactions with GEFs, GAPs, and effector proteins. Many Rabs link directly or indirectly to motor proteins (myosin, dynein, and kinesin) and their associated adaptors (4). Rab connections to the cytoskeleton control processes such as vesicle trafficking, organelle structure, and dynamics (2,5). However, the molecular basis for phosphodependent control of membrane trafficking by Rab GTPases is poorly understood.

We have recently described a novel X-cap motif that enables the RILP family of effectors to bind to phosphorylated

Submitted January 27, 2021, and accepted for publication March 3, 2021.
*Correspondence: khan@crystal.harvard.edu
Editor: Wendy Shaw.
https://doi.org/10.1016/j.bpj.2021.03.030
© 2021 Biophysical Society.
This is an open access article under the CC BY license (http://creativecommons.org/licenses/by/4.0/).

Rab GTPases (6). The N-terminal RH1 (RILP homology) domain of the cytosolic adaptor Rab-interacting lysosomal protein-like 2 (RILPL2) binds to the globular tail domain of myosin Va (7). The C-terminal RH2 domain of RILPL2 encodes a Rab-binding domain (RBD) that adopts a parallel α-helical coiled-coil structure. The N-terminus of the RBD forms an X-cap motif that resembles a hook that winds around its partner. This motif is stabilized by a short antiparallel β-strand-like interaction and has an overall X-shaped conformation that caps the α-helical coiled coil. In the structure of pRab8a with a minimal RBD (residues 129–165), the X-cap enables projection of an Arg residue ($R132^{RL2}$) from each monomer toward a phosphorylated threonine residue of Rab8a ($pT72^{R8}$). In cells, the kinase LRRK2 phosphorylates Rab8a and Rab10 at conserved threonine residues in the switch 2 region, and the post-translational modification tunes the strength of Rab interactions with their binding partners (8). Rab8a/10 phosphorylated at pT72/pT73 recruit RILPL2 to membranes and affect the growth of primary cilia in model cell lines (9). In previous work, we also showed that myosin Va binding to RILPL2 enhances its affinity for pRab8a through an unknown mechanism (6). LRRK2 is the leading cause of inherited Parkinson's disease, a disorder of the central nervous system that manifests as a progressive degeneration of motor mobility, balance, and tremors. Phosphodependent recruitment of RILPL2 by pRab8a/10 is a part of the underlying molecular pathways that connect LRRK2 to neuronal degeneration (10). Therefore, it is critical to understand the biophysical processes that underpin Rab regulatory pathways associated with Parkinson's disease.

Here, we have determined the structure of pRab8a in a complex with an extended RBD of RILPL2 (117–165; abbreviated as $RL2^{117}$) at 1.9-Å resolution. The structure includes residues preceding the X-cap that were not mapped in our previous work. In addition, we have determined the structure of the phosphomimetic T72E mutant of Rab8a ($Rab8^{TE}$) in complex with the minimal RBD of RILPL2 (129–165; $RL2^{129}$) at 1.7-Å resolution. These two structures are analyzed in comparison to the previously published structure of pRab8a in complex with the minimal RBD of RILPL2 (129–165; $RL2^{129}$). We find that the overall structures are identical in all three complexes with no significant differences in intermolecular contacts. However, there are dramatic conformational changes localized to two arginine residues ($R130^{RL2}$, $R132^{RL2}$) in the X-cap of RILPL2. The complex with the extended RBD ($RL2^{117}$) has two well-ordered stacked arginines in which R130 appears to stabilize the conformation of R132, which directly forms a salt bridge to the $pT72^{R8}$. The other two complexes display various levels of disorder, suggesting an influence of the upstream residues of RILPL2 on pRab8a recognition. Calorimetry reveals that $RL2^{117}$ has a significantly higher affinity for pRab8a relative to $RL2^{129}$. We conclude that a robust interaction between the RILP family of effectors with cognate phospho-Rabs is dependent on a dual arginine recognition motif. Mutation of distal R130 to lysine/glutamine is sufficient to abolish interactions with pRab8a in cells. The apparent sensitivity of the dual arginine motif to the upstream region of RILPL2 suggests a mechanism by which myosin:RILPL2 complex formation may regulate its recruitment to phospho-Rab8a membranes.

MATERIALS AND METHODS

Escherichia coli expression constructs

The *E. coli* expression optimized cDNA for Rab8a was obtained from Genscript and covers the residues 1–181 of human Rab8a (Q67L; Q67L + T72E), as previously described (6). The Q67L mutation maintains the Rab8a in the active GTP-bound conformation. The cDNA was cloned into the pET28a vector using *Nde*I/*Bam*HI cloning sites and provides a hexahistidine tag that is cleavable by thrombin. The cDNA for human $RILPL2^{117-165}$ was amplified by PCR from a human full-length RILPL2 construct in pET28a (6) using the following primers: 5′-C CAG GGA GCA GCC TCG GGC CCG AAC AAG ATG GTG G-3′ (forward); 5′-GC AAA GCA CCG GCC TCG TTA ACC GCT TTT GTA GCA TTG CAG-3′ (reverse). Similarly, the cDNA for human $RILPL2^{129-165}$ was made using the following primers: 5′-C CAG GGA GCA GCC TCG AAC CGT CCG CGT TTC ACC C-3′ (forward); 5′-GC AAA GCA CCG GCC TCG TTA ACC GCT TTT GTA GCA TTG CAG-3′ (reverse). The resulting DNA was cloned using ligation-independent methods into the pLIC-MBP vector (11). The construct was sequenced and transformed into *E. coli* BL21(DE3) for expression as His_6-MBP-(TEV)-$RILPL2^{117-165}$.

Protein production

Expression of Rab8a was carried out in LB (Miller) broth supplemented with antibiotic (34 μg/mL kanamycin or 100 μg/mL ampicillin) at 37°C. Media, chemicals, and antibiotics were obtained from FORMEDIUM. At an OD_{600} of 0.6, the culture was cooled to 18°C for ~1 h and induced with 0.5 mM IPTG, after which cells were grown overnight at 18°C. Expression of His_6-MBP-$RILPL2_{117\text{-}165}$ was carried out in LB (Miller) medium supplemented with 100 μg/mL ampicillin (FORMEDIUM) at 37°C. At an OD_{600} of 0.6–0.8, the culture was induced with 0.5 mM IPTG. Cells were grown for an additional 4 h at 37°C. After induction, cells were harvested by centrifugation, and the pellets were resuspended in His-tag extraction buffer (20 mM Tris-HCl, 300 mM NaCl, 10 mM imidazole, and 10 mM β-mercaptoethanol (pH 8.0); +5 mM $MgCl_2$ for Rab constructs). Cells were lysed by sonication, and the cell lysate was centrifuged at 26,000 × *g* for 45 min at 4°C to remove cellular debris. The supernatants were loaded onto an Ni^{2+}-agarose resin (Qiagen). The resin was washed with a 10-fold excess of extraction buffer followed by washing with a five-fold excess using the same buffer supplemented with 40 mM imidazole before elution of the bound proteins using extraction buffer supplemented with 200 mM imidazole. The eluted proteins were dialyzed against 20 mM Tris-HCl, 100 mM NaCl, 5 mM $MgCl_2$, and 1 mM DTT (pH 7.5). Overnight incubation at 4°C with thrombin (GE Healthcare) or TEV protease was used to remove the N-terminal hexahistidine or hexahistidine-MBP tags from each protein. After cleavage, the proteins were run through a second Ni^{2+}-agarose column. The flow-through fractions were collected, while the uncut proteins remained bound to the resin. The peptide corresponding to residues 129–165 of RILPL2 was synthesized with an N-terminal hexahistidine tag (GenScript). The peptide was solubilized in aqueous buffer (20 mM Tris-HCl, 100 mM NaCl, 5 mM $MgCl_2$, 1 mM DTT (pH 7.5)) before crystallization trials.

The second nickel procedure was repeated to minimize the amount of uncleaved His_6-MBP-RILPL2 precursor. Cut $RL2^{117}$ was dialyzed into

low-salt buffer (10 mM MES, 10 mM NaCl, 1 mM DTT (pH 6.0)). The protein was then loaded onto a MonoS cation exchange column (GE Healthcare). The column was subjected to a gradient from low-salt buffer into a high-salt buffer (10 mM MES, 1 M NaCl, 1 mM DTT (pH 6.0)) over a 30-mL volume. The collected fractions were analyzed by SDS-PAGE to ensure purity of $RL2^{117}$.

After removal of the hexahistidine tag, Rab8a was further purified by running the protein through a Superdex 75 (16/60) gel filtration column (GE Healthcare) equilibrated in column buffer (20 mM Tris-HCl, 100 mM NaCl, 5 mM $MgCl_2$, 1 mM DTT (pH 7.5)). The peak fraction containing pure protein was collected and concentrated before crystallization and biophysical experiments. The phosphorylation of Rab8a by MST3 kinase and its specificity to T72 have been described in detail (6). In brief, Rab8a was mixed with MST3 at an 8:1 molar ratio, and the buffer was adjusted to the following conditions: 50 mM Tris-HCl, 150 mM NaCl, 10 mM $MgCl_2$, 2 mM ATP (pH 7.5). Phosphorylation took place at room temperature overnight. The phosphorylation mixture was dialyzed against low-salt buffer and loaded to a MonoS (GE Healthcare) column. Phosphorylated Rab8a was separated from unphosphorylated Rab8a by ion exchange chromatography by a 50% gradient from a 10 mM to 1 M salt buffer (10 mM MES, 5 mM $MgCl_2$, 1 mM DTT (pH 5.2)). The phosphorylation of Rab8a was confirmed by PhosTag gel electrophoresis before subsequent experiments.

Crystallization, data collection, and refinement

Crystals of $Rab8^{TE}$:$RL2^{129}$ complex were obtained in a 1:2 molar ratio of protein/peptide at a total of 12 mg/mL. Crystals were grown in 100 mM HEPES buffer (pH 7), 10% PEG 4000, and 10% 2-propanol. Plate-like crystals were harvested in precipitant supplemented with 25% glycerol and stored frozen in liquid nitrogen. X-ray data were collected under a cryogenic nitrogen stream at 100 K (beamline 24-ID-C; Advanced Photon Source). Crystals of pRab8(Q67L):$RL2^{117}$ complex were obtained in a 1:1 molar ratio of protein/peptide at a total concentration of 5 mg/mL. Crystals were grown in 150 mM DL-Malic acid supplemented with 20% PEG3350. Rod-like crystals were harvested in precipitant supplemented with 25% glycerol and stored frozen in liquid nitrogen. X-ray data were collected under a cryogenic nitrogen stream at 100 K (beamline 24-ID-C; Advanced Photon Source).

Native diffraction data were reduced using XDS and Aimless, followed by structure determination using the Phaser software in the PHENIX package (12,13). Structures were solved using Rab8a (Protein Data Bank, PDB: 4lhw) (14) and RILPL2 (PDB: 6rir) as search models. Because the $Rab8^{TE}$:$RL2^{129}$ crystals are isomorphous to the previously determined pRab8a:$RL2^{129}$ structure (6), identical reflections were flagged for the R-free subset. Refinement was performed using multiple rounds of model building and energy minimization using PHENIX and COOT (15). The asymmetric unit contains two molecules of $Rab8^{TE}$ (chain A: 2–177; B: 4–176) bound to GTP and two molecules of the RILPL2 (chain D: 129–159; E: 129–160). The structure of pRab8a:$RILPL2^{117-165}$ was solved and refined in a similar manner. However, the space group is different, and the asymmetric unit consists of one molecule of pRab8 and one molecule of $RL2^{117}$. Details of data collection, structure refinement, and deposited PDB files are shown in Table 1. Unless indicated, structures were typically aligned using the secondary structure matching algorithm implemented in COOT. The C_α atoms of (p)Rab8a in the various structures typically aligned with a root mean-square displacement (RMSD) of 0.4–0.6 Å.

Pulldowns and isothermal titration calorimetry

For in vitro pulldowns, hexahistidine-tagged full-length RILPL2 (1–211) or hexahistidine-MBP-tagged truncated RILPL2 constructs were used. Rabs and RILPL2 constructs (10 μM each) were mixed together in 1.5-mL centrifuge tubes with 25 μL Ni^{2+}- agarose resin in a final volume of 1 mL of binding buffer (20 mM Tris (pH 8.0), 300 mM NaCl, 20 mM imidazole, 5 mM $MgCl_2$, 10 mM β-mercaptoethanol). The reaction mixture was mildly shaken for 15 min followed by gentle centrifugation (1000 rpm). The resin was washed three times with 1 mL of the binding buffer. After release of proteins from resin with 50 μL elution buffer (20 mM Tris-Cl (pH 8.0),

TABLE 1 Crystallographic data and refinement statistics

	$Rab8^{TE}$	*$Rab8^{TE}$:RILPL2* (129–165)	*pRab8a:RILPL2* (117–165)
Beamline	NSLSII FMX	NECAT APS, 24-ID-C	NECAT APS, 24-ID-E
Wavelength (Å)	0.9789	0.9789	0.97918
Space group	P 2_1	P 2_1 2_1 2_1	C 2 2 2_1
Asymmetric unit	2× Rab8a	2× Rab8a, 2× RILPL2	1× Rab8a, 1× RILPL2
Cell (a, b, c, Å)	36.09, 118.38, 39.6	60.698, 71.733, 116.39	62.773, 68.43, 128.1
(α,β,γ, °)	90, 101.96, 90	90, 90, 90	90, 90, 90
Resolution (Å)	29.29–1.72 (1.76–1.72)	61.07–1.684 (1.744–1.684)	46.26–1.9 (1.968–1.9)
Number of reflections: total (unique)	144,140 (32,822)	325,450 (10,484)	183,379 (15,819)
Completeness (%)	96 (85.2)	96.86 (79.50)	96.02 (90.3)
$<I/\sigma>$	6.9 (2.6)	20.31 (2.44)	16.79 (0.35)
Multiplicity	4.4 (3.9)	5.7 (2.3)	8.6 (7.9)
Rmerge	0.135 (0.441)	0.0543 (0.403)	0.05713 (4.301)
$CC_{1/2}$	0.986 (0.806)	0.998 (0.584)	0.999 (0.54)
**Refinement**			
R_{work}	0.1865 (0.2184)	0.1815 (0.3305)	0.2187 (0.5266)
R_{free}	0.2302 (0.2690)	0.2028 (0.3577)	0.2669 (0.5685)
R-free test size	1632 (163)	2867 (217)	1040 (111)
RMSD bond lengths (Å)	0.006	0.011	0.014
RMSD bond angles (°)	0.86	1.32	1.33
Average overall *B*-factor	19.83	24.93	57.94
Mean B-factors ($Å^2$) protein/GTP/waters	18.92/14.64/29.12	23.44/14.15/36.21	58.28/48.17/53.94
Ramachandran analysis favored/allowed (%)	97.96/2.04	96.53/2.72	97.17/2.83
PDB accession code	6whe	6sq2	7lwb

Values in parentheses correspond to the statistics in the highest resolution bin. $R_{merge} = \Sigma_{hkl}\ \Sigma_j |I_{hkl,j} - <I_{hkl}>| / \Sigma_{hkl}\ \Sigma_{jhkl,j}$. $R_{work} = \Sigma_{hkl}|F_{o,hkl} - F_{c,hkl}| / \Sigma_{hkl}F_{o,hkl}$.

300 mM NaCl, 200 mM imidazole), samples were subjected to SDS-PAGE and visualization with 0.5% Coomassie Brilliant Blue.

Calorimetry experiments were performed in triplicate. The N-terminal polyhistidine-tagged RILPL2 peptide (His$_6$-RILPL2$^{129-165}$), or recombinantly expressed RILPL2$^{117-165}$, was dialyzed together with pRab8a/Rab8TE (20 mM Tris-HCl, 150 mM NaCl, 5 mM $MgCl_2$, and 1 mM DTT (pH 7.5)). Protein concentrations were calculated based on their A_{280} using an ND-1000 NanoDrop spectrophotometer (Thermo Scientific). Samples were centrifuged at 13,200 rpm for 10 min before the determination of protein concentration and ITC analyses. Injection of Rab8^{TE}/pRab8a into RILPL2 was performed, rather than the other way around. RILPL2 injections into buffer revealed a large heat of dilution, which complicated estimates of binding enthalpy and K_d. The concentrations of proteins for injections were between 400 and 600 μM Rab8a and 40–60 μM RILPL2. Data were processed using Origin 7.0 with the ITC plug-in.

Plasmids for cellular assays

The plasmids used for coimmunoprecipitation experiments were acquired from MRC PPU Reagents and Services (https://mrcppureagents.dundee.ac.uk/reagents-proteins/overview): GFP-empty pcDNA5 FRT/TO (DU13156); Flag-LRRK2 Y1699C pCMV (DU13165); HA-Rab8a WT pCMV (DU35414); RILPL2-GFP WT pcDNA5D FRT/TO (DU27481); RILPL2-GFP P128A pcDNA5D FRT/TO (DU68411); RILPL2-GFP D127A pcDNA5D FRT/TO (DU68427); RILPL2-GFP D127N pcDNA5D FRT/TO (DU68428); RILPL2-GFP T126A pcDNA5D FRT/TO (DU68412); RILPL2-GFP D124N pcDNA5D FRT/TO (DU68429); RILPL2-GFP D124A pcDNA5D FRT/TO (DU68413).

Antibody reagents

Antibodies used in this study were diluted in 5% w/v bovine serum albumin in TBS supplemented with 0.1% Tween-20 (TBS-T) and 0.03% w/v sodium azide. The rabbit monoclonal antibody for total LRRK2 (N-terminus) was purified at the University of Dundee (16). Anti-GFP (PABG1; Chromotek, used at 1:1000), anti-HA (3F10; Merck, used at 1:1000), anti-pT72-Rab8a (MJF-R20; Abcam, used at 0.5 μg/mL), anti-LRRK2 C-terminal (N241A/34; Neuromab, used at 1:1000), and anti-α-tubulin (3873S; CST, used at 1:5000). Secondary antibodies used were LI-COR IRDye for 800CW goat anti-rabbit (925–32211), goat anti-mouse (926–32210), and 680LT goat anti-rat (925–68029) and goat anti-mouse (926–68020), all used at 1:10,000 dilution in TBS-T.

Culture and transfection of cells

HEK293 cells were cultured in Dulbecco's modified Eagle medium (Glutamax; Gibco) supplemented with 10% fetal bovine serum (Sigma), 100 U/mL penicillin, and 100 μg/mL streptomycin. Transient transfections were performed 40–48 h before cell lysis using polyethylenimine PEI (Polysciences) at around 60–70% confluence. Transfections for coimmunoprecipitation experiments were done in 10-cm round cell culture dishes using 3 μg of Flag-LRRK2 Y1699C, 1 μg of HA-Rab8a, and 1 μg of GFP control or RILPL2-GFP cDNA construct per dish diluted in 1 mL of OPTIMEM media and supplemented with 20 μg of PEI, incubated for 20 min before being added to the cell media. At 90 min before lysis, cells were treated with 500 nM of MLI-2 inhibitor or 0.1% DMSO control.

Coimmunoprecipitation of Rab8a and RILPL2

Cells were washed with phosphate-buffered saline and lysed in lysis buffer—50 mM Tris-HCl (pH 7.5), 1 mM EGTA, 10 mM sodium β-glycerophosphate, 50 mM sodium fluoride, 5 mM sodium pyrophosphate $\times$ 10H_2O, 0.27 M sucrose—and supplemented fresh before lysis with 1% v/v Triton-x100, one tablet of cOmplete Mini (EDTA-free) protease inhibitor (11836170001; Merck) per 10 mL of buffer, 0.1 μg/mL of microcystin, and 1 μM of sodium orthovanadate. Lysates were clarified by centrifugation at 17,000 $\times$ g for 10 min. For GFP immunoprecipitation, lysates were incubated with nanobody α-GFP binder Sepharose from MRC PPU Reagents and Services for 1 h at 4°C (15 μL of packed resin/0.5 mg of lysate). Bound complexes were recovered by washing the beads three times with wash buffer (50 mM Tris-HCl (pH 7.5), 150 mM NaCl) before eluting with 2$\times$ SDS-PAGE sample buffer supplemented with 1% v/v 2-mercaptoethanol. The samples were denatured at 70°C for 10 min, and the resin was separated from the sample by centrifugation through a 0.22-μm Spinex column (CLS8161; Sigma). Samples were subjected to immunoblotting.

Gel electrophoresis and immunoblot analysis

Samples were run on gels consisting of a 4% w/v acrylamide stacking gel (4% w/v acrylamide, 0.125 M Tris-HCl (pH 6.8), 0.2% v/v tetramethylethylenediamine, and 0.08% w/v ammonium persulfate (APS)) and 10% w/v acrylamide separating gel (10% w/v acrylamide, 0.375 M Bis-Tris (pH 6.8), 1% v/v tetramethylethylenediamine, and 0.05% w/v APS) in MOPS buffer (50 mM MOPS, 50 mM Tris, 1 mM EDTA, 0.1% w/v SDS) at 90–120 V. Proteins were electrophoretically transferred onto nitrocellulose membranes (Amersham Protran 0.45 μm nitrocellulose; GE Healthcare) at 90 V for 90 min on ice in transfer buffer (48 mM Tris/HCl, 39 mM glycine, 20% v/v methanol). Transferred membranes were blocked with 5% w/v nonfat dry milk dissolved in TBS-T (20 mM Tris/HCl (pH 7.5), 150 mM NaCl, and 0.1% v/v Tween 20) at room temperature for 1 h. Membranes were then incubated with primary antibodies overnight at 4°C. After washing membranes in TBS-T 3 $\times$ 15 min, membranes were incubated with secondary antibodies at room temperature for 1 h. After washing membranes in TBS-T 3 $\times$ 15 min, membranes were scanned using LI-COR Odyssey CLx. Protein band intensities were analyzed using LI-COR Image Studio Lite software.

RESULTS AND DISCUSSION

Structural comparisons of Rab8a complexes with RILPL2

The structure of GTP-bound pRab8(Q67L) in complex with RL2^{117} forms crystals with one molecule of pRab8a and one molecule of RILPL2 as the repeating unit. The biological heterotetramer is generated by a twofold crystallographic axis down the length of the central parallel α-helical dimer of RL2^{117}. In the biological assembly, the effector bridges two molecules of Rab8 via hydrophobic and polar interactions. The X-cap is formed at the top of the α-helices, and it is critical for positioning the effector to enable direct contacts between R132^{RL2} and pT72^{R8}. The structure resembles the reported complex of pRab8a in complex with RL2^{129} (Fig. 1 *B*), in which the asymmetric unit was the biologically relevant heterotetramer (6). In this previous structure with the minimal RBD, the salt bridge between pT72^{R8} and R132^{RL2} was well ordered, but the distal R130^{RL2} displayed considerable flexibility. In contrast, the structure of pRab8a in complex with RL2^{117} reveals a well-ordered R130^{RL2} that stacks against R132^{RL2} (Fig. 1 *C*). This stable arrangement of the dual arginines may enable formation of a symmetric twofold crystallographic axis. In the ensuing discussions, stacking will refer to planar π-π interactions of the guanidino side chains of arginines. Crystallographic details are shown in Table 1.

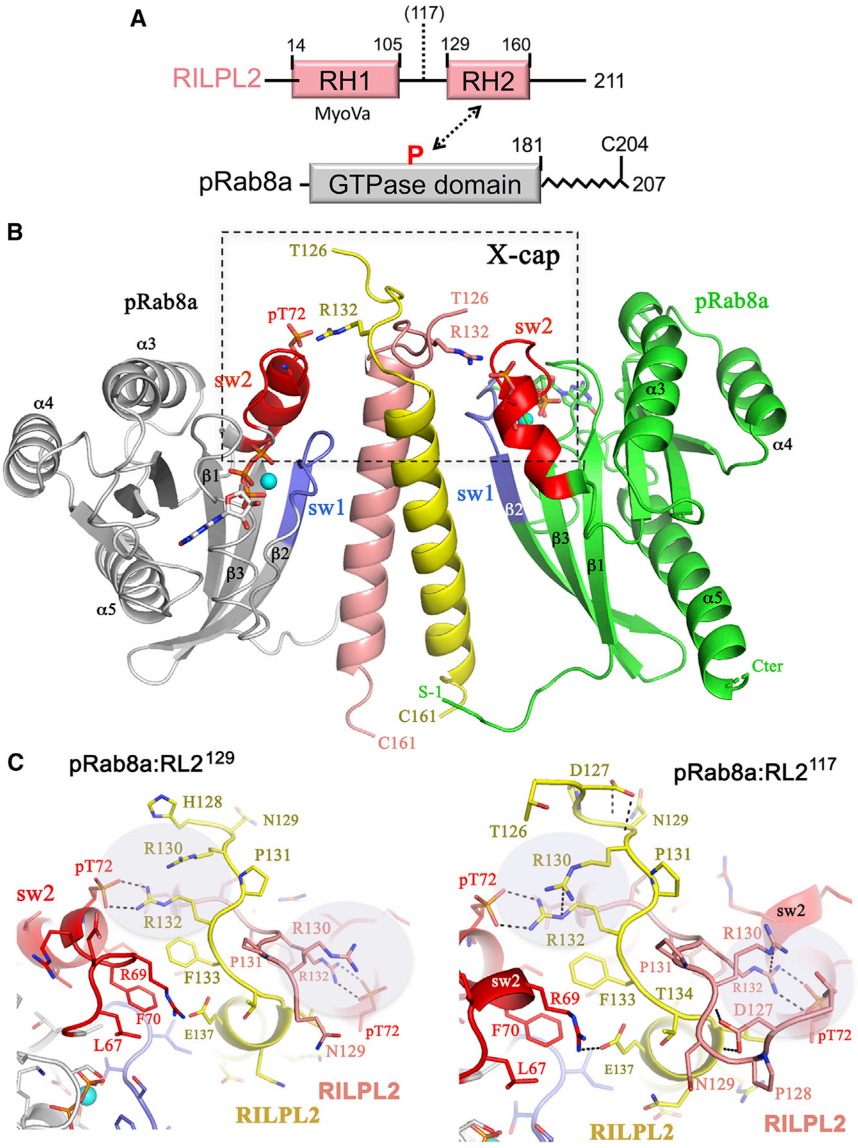

FIGURE 1 Structure of pRab8a:RL2^{117} complex reveals Arg/Arg stacking. (*A*) Domain organization of RILPL2 and Rab8a is shown. The N-terminal RH1 domain of RILPL2 binds to myosin Va. The hinge region between RH1 and RH2 domains is predicted to be flexible from secondary structure analyses. (*B*) Overall view of the pRab8a:RL2^{117} heterotetramer is shown. Dashed rectangle is the X-cap that is seen up close in (*C*) and (*D*). Cyan sphere is an Mg^{2+} ion that interacts with GTP and switch 1 of pRab8a. (*C*) Left: structure of pRab8a:RL2^{129} in the region of X-cap. H128^{RL2} is a non-native residue that comes from the hexahistidine affinity tag. Right: structure of pRab8a:RL2^{117} at the X-cap is shown. R130^{RL2} stacks against the guanidino group of R132^{RL2}. Comparisons of arginine conformations at the interfaces are highlighted with the shaded circle for complexes in (*B*) and (*C*). To see this figure in color, go online.

Although the RBD of the complex begins at 117, residues 117–125 are disordered. The model contains residues 126–161 of RILPL2 and resembles closely the minimal RBD (129–161) of the previous structure (pRab8a:RILP^{129} complex). There are no additional interactions with pRab8a, and upon superposition, equivalent backbone residues have a root mean-square deviation of 1.44 Å. The only significant difference between the complexes is the conformation of R130^{RL2}, which is discussed in more detail below. As a further comparison, the structure of a phosphomimetic mutant Rab8a-T72E (Rab8^{TE}) in complex with RILP^{129} has also been determined. The complex Rab8^{TE}:RILP^{129} crystallizes in the same space group as pRab8a:RILP^{129} (Table 1), and again, there are no significant differences at the interface.

The three complexes afford an excellent opportunity to investigate the structural and thermodynamic details of phospho-Rab binding by effectors. Electron density maps reveal that one side of the E72^{R8}:R132^{RL2} interaction in the Rab8^{TE}:RL2^{129} complex is well ordered and mimics the pT72^{R8}:R132^{RL2} salt bridge. However, the opposite side shows some flexibility as evidenced by the poor density and geometry (Fig. 2 *A*). In contrast, the key pT72^{R8}:R132^{RL2} interaction in the pRab8a:RILP^{129} complex is well ordered, suggesting a stronger salt bridge with phosphothreonine (Fig. 2 *B*). There is also partial stacking of the R132/R130 side chains in the two complexes with the minimal (RILP^{129}) RBD. R130^{RL2} in the complex pRab8:RILP^{129} reveals better stacking relative to Rab8^{TE}, but there remains considerable flexibility as evidenced by electron density maps (Fig. 2 *B*).

Strikingly, the newly determined structure of pRab8a:RL2^{117} reveals an ordered R130 side chain that forms strong stacking interactions with the guanidino group of R130 (Fig. 2 *C*). As a measure of the close contacts, the

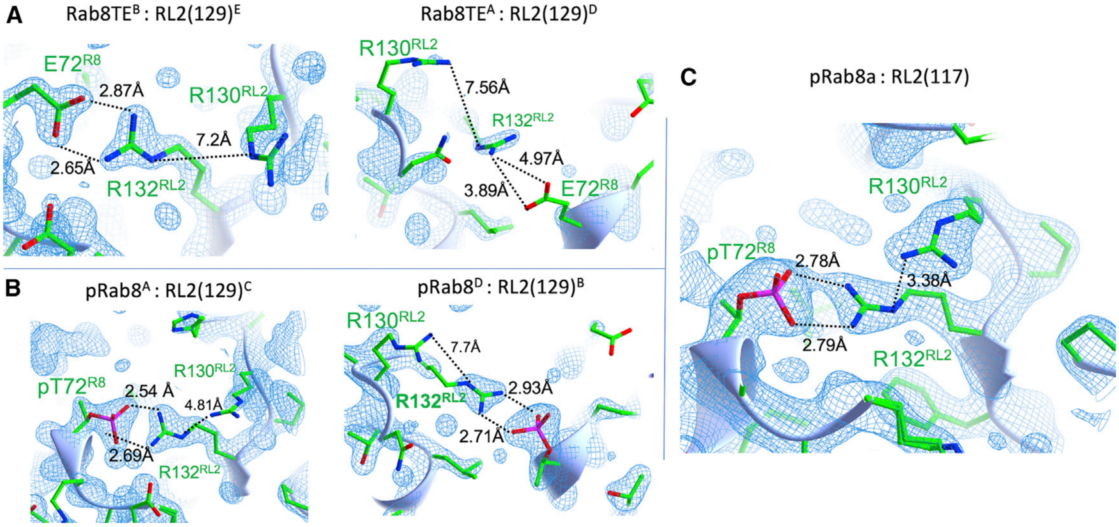

FIGURE 2 Electron density at the phosphate recognition motif of RILPL2. The contour level for all figures is at 1.5σ. The asymmetric unit is the physiological 2:2 complex for (*A*) and (*B*), whereas the pRab8:RL2^{117} is a 1:1 complex in the crystal. RL2, RILPL2; R8, Rab8a. (*A*) Complex Rab8^{TE}:RL2^{129} reveals disorder and poor contacts for one of the E72^{R8}:R132^{RL2} interactions. (*B*) For the complex pRab8a:RL2^{129}, both pT72^{R8}:R132^{RL2} contacts are well ordered. However, the R130 side chain is flexible. (*C*) In the pRab8a:RL2^{117} complex, both R130^{RL2} and R132^{RL2} are well ordered and are involved in guanidino stacking interactions. To see this figure in color, go online.

distance between R130(Nε) and R132(NH2) is 3.4 Å. In contrast to the other complexes, pRab8a:RILP^{117} forms a symmetric twofold axis down the length of the coiled coil. Therefore the two salt bridges between pT72 and R132 are identical by symmetry in the biological heterotetramer. Rab8^{TE}:RL2^{129} and pRab8a:RL2^{129} assemble as heterotetramers in the lattice. As suggested previously, it is possible that conformational flexibility in R130/R132 may prevent a symmetric twofold axis from forming in the other two complexes. Previously, we reported that R130^{RL2} was essential for complex formation in cells. In the context of full-length RILPL2, even a mutation to lysine (R130K) significantly reduced the interaction with pRab8a (6). However, the contribution of this distal arginine to complex formation was unclear because the side chain was relatively disordered. Given the short RBD (129–165) in previous work, we could not exclude the possibility that both R130 and R132 might directly interact with pT72^{R8} in longer variants of RILPL2. Here, the structure of pRab8a with an extended RBD–RL2^{117} suggests that R130^{RL2} contributes to π-π stacking interactions with R132^{RL2} and stabilizes its salt bridge with pT72^{R8}. A lysine residue (R130K) cannot substitute for this interaction, thus providing a rationale for the inability of a positive charge to maintain binding to pRab8a.

Thermodynamics of complex formation

In vitro pulldowns were performed to qualitatively assess the affinities of the three complexes (Fig. 3 *A*). These experiments suggested that full-length RILPL2 (1–211) has less affinity to pRab8a relative to the truncated variant RL2^{117}. Although the in vitro reduction in affinity appears to be modest, the finding is surprising because effectors are modular and RBD interactions with Rabs are generally independent of other domains (5). Isothermal titration calorimetry (ITC) was then exploited to provide more insight into the binding affinities (Fig. 3 *B*; Table 2). The data reveal that the affinity between pRab8a and RL2^{117} is significantly stronger than other complexes, with a more robust enthalpy contributing to complex formation (Fig. 3 *B*). As discussed previously, comparisons of the three complexes—Rab8TE:RL2^{129}, pRab8:RL2^{129}, and pRab8:RL2^{117}—reveal no significant differences apart from the side chain of R130. Therefore, the increased enthalpic contribution to the binding affinity can be attributed to a stronger salt bridge between pT72^{R8} and R132^{RL2} (Fig. 3 *C*). Although the distal R130^{RL2} is relatively far from the nearest phosphate oxygen (5.6 Å), it may also contribute to the enthalpy through long-range electrostatic interactions. The bar graph of free-energy signatures (ΔH, TΔS) also indicates that the entropic term contributes favorably toward formation of all of the complexes. However, the enthalpic gains are countered by a reduced entropic contribution, which can partially be attributed to ordered guanidino side chains. The complexes Rab8^{TE}:RL2^{129} and pRab8:RL2^{129} have a similar K_d, but favorable enthalpy for the pT72:R132 interaction is offset by a reduction in the entropic gains (Fig. 3 *D*; Table 2). Intriguingly, relative to the truncated RL2^{117} variant, full-length RILPL2 has a reduced affinity to pRab8a with less favorable enthalpy. The N-terminal RH1 domain appears to antagonize the interactions between pRab8 and the RH2 domain by an unknown mechanism that requires further investigation.

In addition to complexes, we also determined the structure of uncomplexed Rab8^{TE}. This was performed to support the hypothesis that thermodynamic parameters can be attributed to complex formation rather than intrinsic structural changes

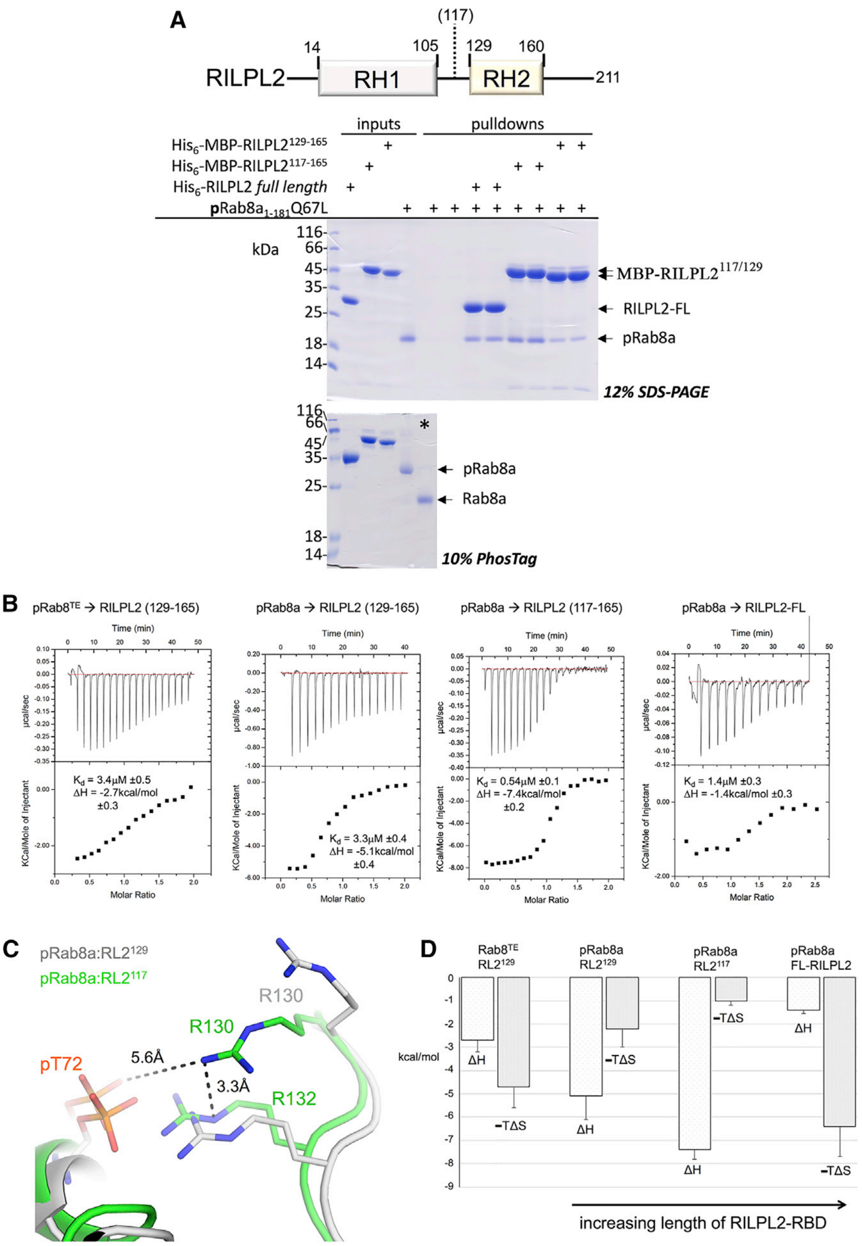

FIGURE 3 Extended RBD of RILPL2 enhances the enthalpic contribution to the binding affinity. (*A*) In vitro pulldown of pRab8a with hexahistidine-tagged RILPL2 variants. Each pulldown was performed in duplicate with 10 μM concentrations of bait and prey proteins. Domain organization of RILPL2 is above the pulldown. Asterisk in the phos-tag control gel is a Rab8a sample to show purity of pRab8a in pulldowns. (*B*) ITC titrations of Rab8a into RILPL2 are shown. Typical concentrations were 400–600 μM pRab8a/Rab8^{TE} and 40–60 mM RILPL2 variants. (*C*) Comparisons of the structures of pRab8a:RILP^{129} and pRab8a:RILP^{117} are shown. The key difference at the interface is the conformation of R132^{RL2}. (*D*) Enthalpy and entropy terms from ITC data (Table 2) are represented as a bar graph. The trends show that increasing enthalpic contributions are balanced by an entropic cost to complex formation. ITC values were calculated from the mean and standard deviations of 3 independent measurements. To see this figure in color, go online.

from the negative charge at switch 2. The structure of Rab8^{TE} reveals no significant conformational differences in the overall fold relative to the complex Rab8^{TE}:RL2^{129} (Fig. S1).

TABLE 2 Thermodynamics of RILPL2 binding to pRab8a/Rab8^{TE}

	K_d (μM)	ΔH (kcal/mol)	−TΔS (cal/k•mol)	N
**Rab8^{TE}(Q67L)**				
RL2^{129}	3.4 ± 0.5	−2.7 ± 0.3	−4.6 ± 0.5	1.03 ± 0.05
**pRab8a(Q67L)**				
RL2^{129}	3.3 ± 0.4	−5.1 ± 0.4	−2.2 ± 0.4	0.92 ± 0.2
RL2^{117}	0.54 ± 0.1	−7.4 ± 0.2	−1 ±0.3	1.1 ± 0.15
RL2-FL	1.4 ± 0.3	−1.4 ± 0.3	−6.4 ± 1.2	1.04 ± 0.1

Similarly, the recent structure of phosphorylated Rab8a at S111 is identical to the unphosphorylated variant (17). Although the structure of uncomplexed pRab8a-pT72 has not yet been determined, it is likely that phosphorylated Rabs do not undergo significant conformational changes upon complex formation with RILPL2.

Mutagenesis and cellular assays

Mutational and cellular analyses of RILPL2 residues preceding R130^{RL2} were performed to assess the contribution of this segment to pRab8a binding (Fig. 4). GFP-tagged full-length RILPL2 variants were overexpressed in HEK293 cells together with HA-tagged Rab8a and Flag-tagged LRRK2

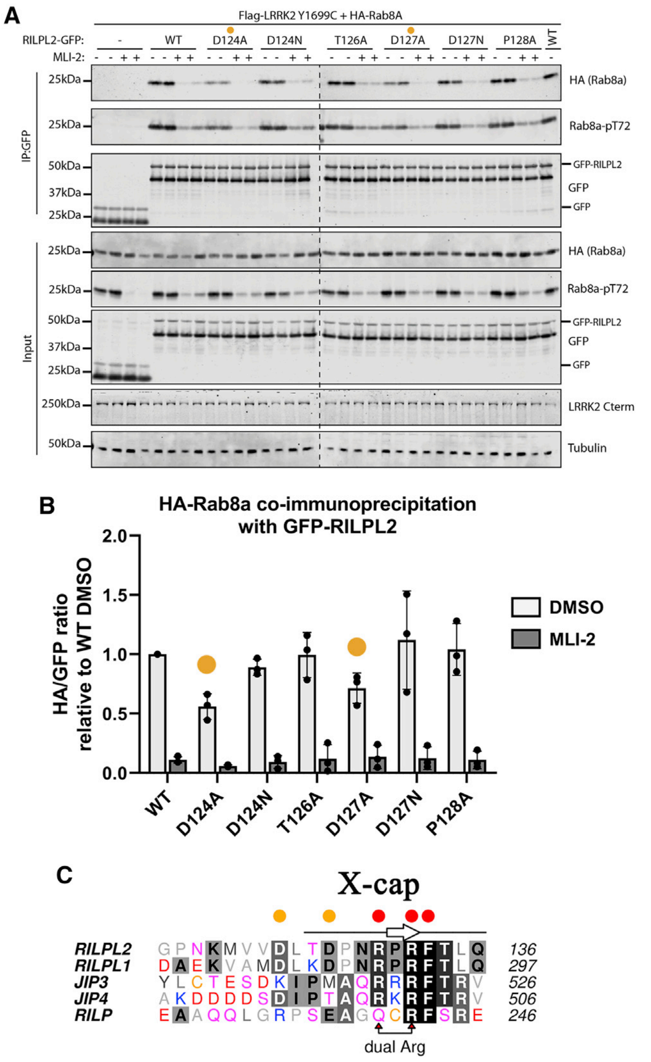

FIGURE 4 Mutational and cellular assays suggest an extended RBD contributes to pRab8a binding. (*A*) Coimmunoprecipitation studies of overexpressed RILPL2 and pRab8a. HEK293 cells were transiently transfected with constructs expressing Flag LRRK2[Y1699C], HA-Rab8a, and WT or mutant RILPL2-GFP. At 48 h post-transfection, cells were treated with ±500 nM MLi-2 for 90 min and then lysed. Upper panel: labeled IP:GFP:RILPL2-GFP was immunoprecipitated using GFP binder Sepharose and immunoprecipitates evaluated by immunoblotting with the indicated antibodies. Immunoblots were developed using the LI-COR Odyssey CLx Western blot imaging system with the indicated antibodies at 0.5–1 μg/mL concentration. Lower panel, labeled input: 10 μg whole-cell lysate was subjected to LI-COR immunoblot analysis. Each lane represents cell extract obtained from a different dish of cells. Similar results were obtained in three separate experiments. (*B*) Quantification of pull-down intensities from multiple repetitions. The yellow circles denote a reduction in binding for mutants. Error estimates are the mean and standard deviation of 3 independent measurements. (*C*) Sequence alignment of the predicted X-cap regions of the phospho-Rab-binding family of effector proteins. Red circles above the sequence of RILPL2 indicate a severe defect on pRab8a binding upon mutagenesis. Yellow circles indicate a partial defect on the binding to pRab8a. Lines and arrow above the alignment correspond to the secondary structure (loop, β-strand) of RILPL2. To see this figure in color, go online.

Y1699C to ensure maximal phosphorylation of Rab8a. LRRK2 specific inhibitor Mli-2 was used at 500 nM concentration for 90 min as a control. RILPL2 was immunoprecipitated using anti-GFP resin, and the samples were subjected to immunoblotting. These studies reveal that single mutants D124A^{RL2} and D127A^{RL2} are partially defective in binding to pRab8a (Fig. 4, *A* and *B*). The side-chain carboxylate of D127^{RL2} is within 3 Å of the backbone NH groups of N129^{RL2} and R130^{RL2}. Although the geometry is not ideal, these interactions may nevertheless contribute to stabilization of the backbone conformation. It would also explain why D127N^{RL2} does not have the same defect as D127A^{RL2} (Fig. 4 *A*). The effects of mutations are mapped as hotspots on a sequence alignment of the predicted X-cap of effector proteins (Fig. 4, *B* and *C*). There are modest sequence similarities in the upstream region preceding R130 within the RILP family. Red circles denote hotspots in which any mutation abolishes binding in cells (explored in our previous publication (6)), whereas yellow circles denote an intermediate phenotype. The molecular basis for the contribution of D124^{RL2} to phospho-Rab recognition is unknown because the segment 117–125 is flexible in electron density maps.

CONCLUSION

Phosphorylation of Rab GTPases has emerged as a regulatory mechanism for membrane trafficking in a variety of contexts (3). The switch 2 α-helix of Rab GTPases is a hotspot for phosphorylation, and its modification is likely to influence subsequent interactions with GAPs, GEFs, and effector proteins. Here, we show that the conformation of arginine residues from RILPL2 involved in pRab8a recognition is sensitive to the region preceding the RBD. In particular, distal R130^{RL2} is flexible, without a stabilizing influence from upstream residues. However, the full-length effector comprising both RH1/RH2 domains dramatically reduces the enthalpy of complex formation, suggesting that the distal R130 is disordered. The structural and thermodynamic studies also suggest that the enthalpic gain from the salt bridge between R132/pT72 is offset by a reduction in favorable entropy arising from the ordering of the guanidino side chains of R130/R132. A video is shown with the three complexes as a trajectory (Rab8TE:RILP^{129} → Rab8TE:RILP^{129} → pRab8:RILP^{117}) using the Morph application in Chimera (Video S1; 18). Despite the lack of any additional interactions, the upstream segment of RILPL2 appears to stabilize the conformations of the two arginines for optimal interactions with pRab8a. Cation-π and π-π interactions that involve aromatic residues are widely known for their stabilizing influence in a variety of contexts. However, arginine clusters are increasingly being recognized for their contribution to protein complex formation (19–21). Conformational dynamics of RILPL2 have also previously been observed in its interactions with Rab36 and myosin Va (22).

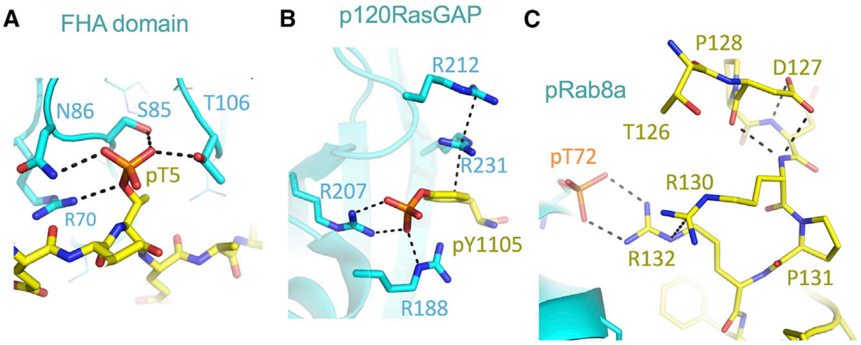

FIGURE 5 Comparisons of the recognition motifs in phosphodependent signaling complexes. Phosphorylated proteins are in yellow, and recognition motifs are cyan. (*A*) Forkhead-associated domain (FHA) in complex with a phosphothreonine peptide (PDB: 1g6g (23)). (*B*) Complex of p120RasGAP with a phosphotyrosine peptide from p190RhoGAP (PDB: 6pxc (24)) is shown. (*C*) Complex of pRab8a:RL2[117] is shown. The recognition motif for pT72[R8] is more peripherally associated relative to other known structures. To see this figure in color, go online.

The structures of numerous signaling complexes involving phosphorylated peptides have been determined. In general, the peptides adopt an extended conformation, and the phosphorylated side chains are deeply embedded, with interactions on both sides of the phosphate moiety (Fig. 5, *A* and *B*). In contrast, the RILPL2 interaction with the phosphorylated switch 2 helix is more peripheral (Fig. 5 *C*). This more subtle interaction motif in the pRab8a:RILPL2 complex may be influenced by the upstream hinge region that connects the RH1 domain to the RH2 domain. We have shown previously that myosin Va binding to the RH1 domain enhances the affinity of the RH2 domain to pRab8a (6). An attractive mechanistic model is that myosin binding facilitates a conformation for the hinge region that enables recognition of pRab8a by a dual arginine motif. Myosin binding to the RH1 domain may enable ordering of the distal arginine (R130), which would enhance the stability of the salt bridge between R132 and pT72 of Rab8a. One prediction from this model is that myosin:RILPL2 complexes are more likely to be recruited to phospo-Rab8a membranes downstream of LRRK2 activation.

SUPPORTING MATERIAL

Supporting material can be found online at https://doi.org/10.1016/j.bpj.2021.03.030.

AUTHOR CONTRIBUTIONS

D.W. and E.P. analyzed data, performed the research, and wrote the article. A.R.K. designed the project, analyzed data, performed research, and wrote the article.

ACKNOWLEDGMENTS

A.R.K. was supported by the Program for Cellular and Molecular Medicine, Boston Children's Hospital. This work is based on research conducted at the Northeastern Collaborative Access Team beamlines, which are funded by the National Institute of General Medical Sciences from the National Institutes of Health (P30 GM124165). The Eiger 16M detector on 24-ID-E is funded by a NIH-ORIP HEI grant (S10OD021527). This research used resources of the Advanced Photon Source, a U.S. Department of Energy (DOE) Office of Science User Facility operated for the DOE of Science by Argonne National Laboratory under Contract No. DE-AC02-06CH11357. This research also used the FMX beamline of the National Synchrotron Light Source II, a U.S. DOE Office of Science User Facility operated for the DOE Office of Science by Brookhaven National Laboratory under Contract No. DE-SC0012704. The work undertaken by EP was supported by the UK Medical Research Council (grant number: MC_UU_12016/2 (D.R.A.)).

REFERENCES

1. Hutagalung, A. H., and P. J. Novick. 2011. Role of Rab GTPases in membrane traffic and cell physiology. *Physiol. Rev.* 91:119–149.
2. Khan, A. R., and J. Ménétrey. 2013. Structural biology of Arf and Rab GTPases' effector recruitment and specificity. *Structure.* 21:1284–1297.
3. Waschbüsch, D., and A. R. Khan. 2020. Phosphorylation of Rab GTPases in the regulation of membrane trafficking. *Traffic.* 21:712–719.
4. Kjos, I., K. Vestre, …, C. Progida. 2018. Rab and Arf proteins at the crossroad between membrane transport and cytoskeleton dynamics. *Biochim. Biophys. Acta Mol. Cell Res.* 1865:1397–1409.
5. Pylypenko, O., H. Hammich, …, A. Houdusse. 2018. Rab GTPases and their interacting protein partners: structural insights into Rab functional diversity. *Small GTPases.* 9:22–48.
6. Waschbüsch, D., E. Purlyte, …, A. R. Khan. 2020. Structural basis for Rab8a recruitment of RILPL2 via LRRK2 phosphorylation of switch 2. *Structure.* 28:406–417.e6.
7. Wei, Z., X. Liu, …, M. Zhang. 2013. Structural basis of cargo recognitions for class V myosins. *Proc. Natl. Acad. Sci. USA.* 110:11314–11319.
8. Steger, M., F. Tonelli, …, M. Mann. 2016. Phosphoproteomics reveals that Parkinson's disease kinase LRRK2 regulates a subset of Rab GTPases. *eLife.* 5:e12813.
9. Steger, M., F. Diez, …, M. Mann. 2017. Systematic proteomic analysis of LRRK2-mediated Rab GTPase phosphorylation establishes a connection to ciliogenesis. *eLife.* 6:e31012.
10. Singh, P. K., and M. M. K. Muqit. 2020. Parkinson's: a disease of aberrant vesicle trafficking. *Annu. Rev. Cell Dev. Biol.* 36:237–264.
11. Cabrita, L. D., W. Dai, and S. P. Bottomley. 2006. A family of *E. coli* expression vectors for laboratory scale and high throughput soluble protein production. *BMC Biotechnol.* 6:12.
12. McCoy, A. J., R. W. Grosse-Kunstleve, …, R. J. Read. 2007. Phaser crystallographic software. *J. Appl. Cryst.* 40:658–674.
13. Adams, P. D., P. V. Afonine, …, P. H. Zwart. 2010. PHENIX: a comprehensive Python-based system for macromolecular structure solution. *Acta Crystallogr. D Biol. Crystallogr.* 66:213–221.
14. Guo, Z., X. Hou, …, A. Itzen. 2013. Intermediates in the guanine nucleotide exchange reaction of Rab8 protein catalyzed by guanine nucleotide exchange factors Rabin8 and GRAB. *J. Biol. Chem.* 288:32466–32474.

15. Emsley, P., B. Lohkamp, …, K. Cowtan. 2010. Features and development of Coot. *Acta Crystallogr. D Biol. Crystallogr.* 66:486–501.

16. Dzamko, N., F. Inesta-Vaquera, …, D. R. Alessi. 2012. The IkappaB kinase family phosphorylates the Parkinson's disease kinase LRRK2 at Ser935 and Ser910 during Toll-like receptor signaling. *PLoS One.* 7:e39132.

17. Pourjafar-Dehkordi, D., S. Vieweg, …, M. Zacharias. 2019. Phosphorylation of Ser111 in Rab8a modulates rabin8-dependent activation by perturbation of side chain interaction networks. *Biochemistry.* 58:3546–3554.

18. Pettersen, E. F., T. D. Goddard, …, T. E. Ferrin. 2004. UCSF Chimera–a visualization system for exploratory research and analysis. *J. Comput. Chem.* 25:1605–1612.

19. Neves, M. A. C., M. Yeager, and R. Abagyan. 2012. Unusual arginine formations in protein function and assembly: rings, strings, and stacks. *J. Phys. Chem. B.* 116:7006–7013.

20. Vernon, R. M., P. A. Chong, …, J. D. Forman-Kay. 2018. Pi-Pi contacts are an overlooked protein feature relevant to phase separation. *eLife.* 7:e31486.

21. Ferrari, L., R. Stucchi, …, S. G. D. Rüdiger. 2020. Arginine π-stacking drives binding to fibrils of the Alzheimer protein Tau. *Nat. Commun.* 11:571.

22. Cao, Q. J., N. Zhang, …, X. D. Li. 2019. The cargo adaptor proteins RILPL2 and melanophilin co-regulate myosin-5a motor activity. *J. Biol. Chem.* 294:11333–11341.

23. Durocher, D., I. A. Taylor, …, M. B. Yaffe. 2000. The molecular basis of FHA domain:phosphopeptide binding specificity and implications for phospho-dependent signaling mechanisms. *Mol. Cell.* 6:1169–1182.

24. Jaber Chehayeb, R., A. L. Stiegler, and T. J. Boggon. 2019. Crystal structures of p120RasGAP N-terminal SH2 domain in its apo form and in complex with a p190RhoGAP phosphotyrosine peptide. *PLoS One.* 14:e0226113–e0226113.

Biophysical Journal, Volume *120*

Supplemental information

Dual arginine recognition of LRRK2 phosphorylated Rab GTPases

Dieter Waschbüsch, Elena Purlyte, and Amir R. Khan

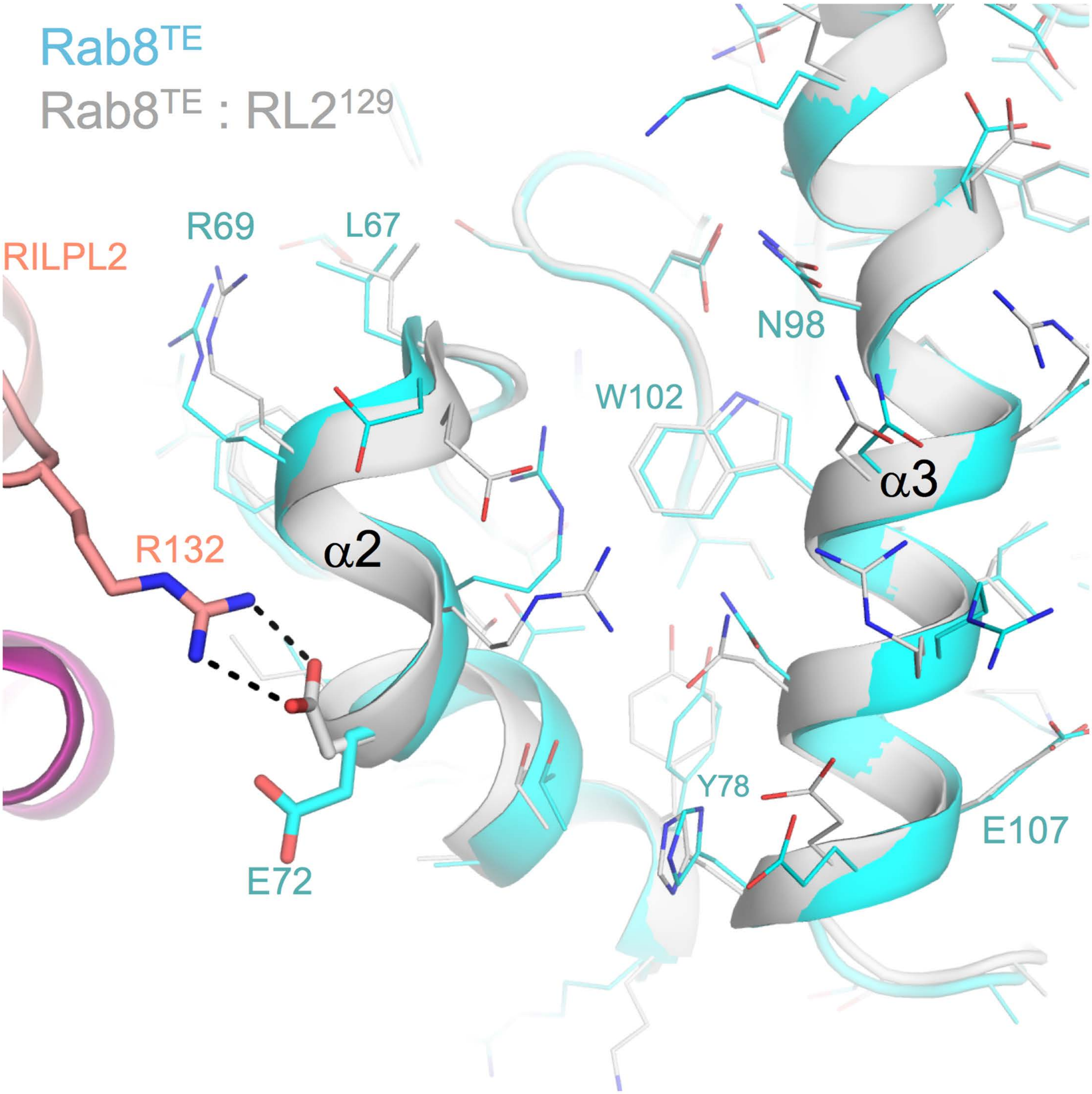

Rab8TE
Rab8TE : RL2129
RILPL2
R69
L67
N98
W102
α3
α2
R132
Y78
E107
E72

Figure S1
